# Sleep Duration and Amyloid β Among Cognitively Healthy Later-Life Adults: A Systematic Review and Meta-Analysis

**DOI:** 10.21203/rs.3.rs-2782680/v1

**Published:** 2023-04-20

**Authors:** Chooza Moon, Aaron Schneiner, Young Eun Cho, Meina Zhang, Hellen Dang, Kelly Vu

**Affiliations:** University of Iowa College of Liberal Arts and Sciences; University of Iowa College of Liberal Arts and Sciences; University of Iowa College of Nursing; University of Iowa College of Liberal Arts and Sciences; University of Iowa College of Liberal Arts and Sciences; University of Iowa College of Pharmacy

**Keywords:** Amyloid plaque, Amyloid beta, sleep, sleep duration, Alzheimer’s disease

## Abstract

**Background:**

Amyloid β (Aβ) is a hallmark of Alzheimer’s disease (AD). Insufficient sleep duration and poor sleep quality have been found to be a risk factor of developing AD because sleep may involve regulating Aβ. However, the magnitude of the relationship between sleep duration and Aβ is still unclear. This systematic review examines the relationship between sleep duration and Aβ in later-life adults.

**Methods:**

We screened 5,005 published articles searched from relevant electronic databases (i.e., PubMed, CINAHL, Embase, and PsycINFO) and reviewed 14 articles for the qualitative synthesis and 7 articles for the quantitative synthesis.

**Results:**

Mean ages of the samples ranged from 63 to 76. Studies measured Aβ using cerebrospinal fluid, serum, and positron emission tomography scans with two tracers: Carbone 11-labeled Pittsburgh compound B or fluorine 18–labeled. Sleep duration was subjectively measured using interviews, questionnaires, or using objective measures such as polysomnography or actigraphy. The studies accounted for demographic and lifestyle factors in their analyses. Five of the 14 studies reported a statistically significant association between sleep duration and Aβ. Using seven eligible articles, our quantitative synthesis demonstrated that the average association between sleep duration and Aβ was not statistically significant (Fisher’s Z = −0.006, 95% CI= −0.065 ~ 0.054).

**Conclusion:**

This review suggests that caution should be taken when considering sleep duration as the primary factor for Aβ levels. More studies are needed using a longitudinal design, comprehensive sleep metrics, and larger sample sizes to advance our understanding of the optimal sleep duration and AD prevention.

## Background

Alzheimer’s disease (AD) is a progressive neurodegenerative disease affecting one in ten adults over the age of 65 worldwide, which poses a considerable economic challenge [1]. More than 6.5 million older Americans suffered from AD in 2022, and the estimated cost for AD is $321 billion worldwide[2]. By the year of 2050, the number of AD in the US is expected to reach 12.7 million individuals. Neurodegenerative processes associated with AD result in the accumulation of senile plaques and pathologic changes in Amyloid β (Aβ) throughout the brain, cerebrospinal fluid (CSF), and serum. [3, 4]. AD biomarkers may be present even decades before clinical AD symptoms appear [5]. However, there are a lack of effective disease-modifying treatments to delay the onset of AD symptoms. Thus, there is a pressing need to identify modifiable risk factors and develop novel interventions to decrease the risk of AD.

Alterations in sleep duration and efficiency can lead to wide-ranging consequences for health and well-being and increase the risk of AD [6]. Current guidelines state that healthy sleep is a sleep duration of 7 or more hours per night for adults between 18 and 60, 7–9 hours for adults between 61 and 64, and 7–8 hours for 65 years and older [7–9]. However, later-life adults who are older than 50 typically experience shorter sleep duration than 7 hours or lower sleep efficiency than 85% than younger adults [10]. In particular, slow-wave sleep declines significantly with age [10]. In addition to the changes in sleep structures, sleep disorders including insomnia and sleep-disordered breathing increase [10]. Additionally, individuals with mild cognitive impairment or AD often experience disruptions in sleep and experience sundown syndrome, but often this condition occurs years prior to impairment [11–13].

Sleep maintains brain and neural homeostasis [14]. During sleep, the brain controls Aβ peptide regulation [15], clears neurotoxins including Aβ plaques [16], and decreases systematic inflammation [17]. Thus, reductions in sleep duration or disruptions during sleep can influence the pathological changes of Aβ. Numerous recent papers and reviews focusing on the overall direction of sleep have suggested that sleep fragmentation or disruption is associated with AD via Aβ or tau pathology [15, 18–21]. These findings suggest that improving sleep efficiency and optimal sleep quantity could be an opportunity to prevent and delay AD pathology by decreasing Aβ deposition and tau hyperphosphorylation. Insomnia, sleep disordered breathing, and sleep fragmentation have been found to be associated with the risk of developing AD and related dementia [6]. However, we do not fully understand what the optimal sleep duration is to prevent AD.

Prior studies speculates that shorter sleep duration can be associated with Aβ levels, because both total and partial sleep deprivation has been shown to increase Aβ levels in plasma [22, 23], CSF [24, 25], or brain [26–28]. For instance, Zhao et al. (2019) found that chronic sleep restriction was associated with increases in Aβ in a mouse model [28]. Furthermore, Kang et al. (2009) suggest that acute sleep deprivation can increase Aβ levels in animals via orexin regulation [29]. To better understand the magnitude of relationship between sleep duration and Aβ, meta-analysis and/or systematic review is needed. However, few meta-analysis and systematic reviews have specifically focused on how sleep duration matters for Aβ accumulation in human studies on adults in later life. The purpose of this systematic review is to focus on the current state of science on how sleep duration is associated with Aβ in the brain, CSF, and serum in older adults.

## Methods

The purpose of this study is to conduct a systematic review to evaluate evidence on how sleep duration is associated with Aβ. The study was registered *a priori* with the International Prospective Register of Systematic Reviews (PROSPERO; registration no. CRD42021266789).

**Figure 1:**
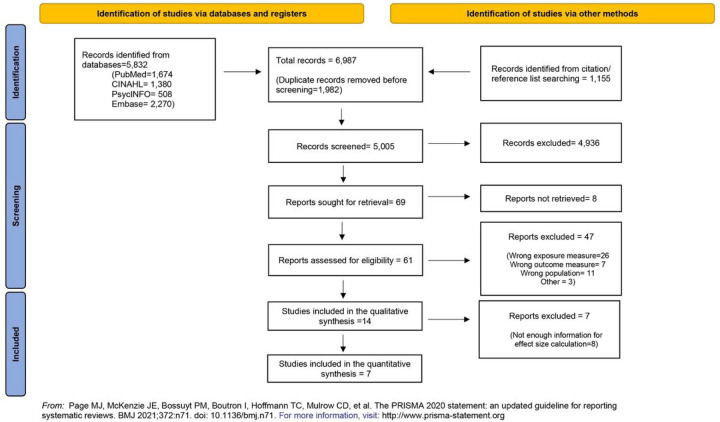
PRISMA 2020 flow diagram

This review was conducted following the guidelines of the Preferred Reporting Items for Systematic Reviews and Meta-Analyses (PRISMA) statement for reporting systematic reviews and meta-analyses [30]. Search strategies were developed with the assistance of a health sciences librarian with expertise in searching articles for systematic reviews. The flow diagram in Fig. 1 provides details on the search strategy and the number of articles each database yielded. Comprehensive strategies, including both index and keyword methods, were devised by the librarian and primary author for the following databases: PubMed, CINAHL (EBSCO platform), Embase (Elsevier platform), and PsycINFO (EBSCO platform). To maximize sensitivity, no pre-established database filters other than the English language filter were used. The full PubMed search strategy, as detailed in the supplemental table A, was also adapted for the other databases. In addition to the database searches, references and cited papers of the 1,156 relevant papers were located using the Scopus database.

Inclusion criteria for the qualitative synthesis were as follows: 1) observational studies with a longitudinal or cross-sectional design, 2) includes exposure variables of sleep duration, 3) has Amyloid β plaques (e.g., Aβ, Aβ_42_, Aβ_40_, Aβ_42_/Aβ_40_) as the outcome, 4) a human study of adults aged ≥ 50 years old, and 5) recruited (or included) cognitively healthy individuals. An additional inclusion criterion for the quantitative synthesis was studies that reported sufficient data for examining the effect sizes, such as Pearson’s correlation (r), means, standard deviations, t, F, or X^2^ values. We excluded studies 1) not written in English, 2) interventional studies, 3) non-peer reviewed papers, proceedings, editorials, and reviews, and 4) the study sample focused only on neurological conditions or sleep disorders. For the quantitative synthesis, we excluded studies that lacked or had inadequate inferential statistical results for calculating the effect size.

The initial search yielded 6,987 articles. After removing 1,982 duplicate articles, a total of 5,005 articles were imported to the web-based systematic review application, Rayyan. Four authors (CM, KV, HD, MZ) screened the abstracts and titles of the 5,005 articles based on the eligibility criteria. Then, additional articles were removed leaving 61 full-text articles that were reviewed by four authors (CM, AS, YC, MZ). A total of 14 articles met the inclusion criteria for the qualitative synthesis and studies met the criteria for quantitative synthesis (Fig. 1). Disagreements were solved through discussion among all authors until consensus was reached.

### Quality analysis

The risk bias of the selected papers was assessed independently by two reviewers using the National Institute of Health Study Quality Assessment Tool (2019) for Observational Cohort and Cross-Sectional Studies (https://www.nhlbi.nih.gov/health-topics/study-quality-assessment-tools). The internal validity of the studies were assessed based on 14 domains: 1) bias due to an unclear purpose, 2) bias due to an unclear specification of the population, 3) bias due to ineligible participants, 4) bias due to recruitment from a different population, 5) bias due to unclear power justification, 6) bias due to measure timing, 7) bias due to time frame, 8) bias due to outcome level, 9) bias due to invalid exposure measure, 10) bias due to frequency of the assessments, 11) bias due to the outcome measure, 12) bias due to an unblinded assessment, 13) bias due to loss during follow up, and 14) bias due to statistical analysis and confounding. For each domain, we categorized the risk of bias as either low or high risk. We rated an item “unclear risk” if there was no information about the risk of bias.

### Statistical analysis

We aggregated the effect sizes across the studies and calculated the publication bias, overall effect sizes, and Q statistics using Comprehensive Meta-Analysis (CMA) version 4 software (Biostat, Inc). We also calculated the effect sizes using Pearson’s r as the effect size index after examining the available information on the correlation between sleep duration and Aβ. The r was extracted from each study and converted to Fisher’s Z = 0.5*Log(1 + Corr)/(1-Corr) [31]. We used a Q statistic to evaluate the heterogeneity of variance. We also calculated the *I*^2^ index using *I*^2^ = 100% × (Q − degree of freedom)/Q to identify how the variance in observed effects reflected the variance in true effects rather than by random error. The random-effects model was applied for the current study because we expect that the sampling distribution varied across the studies and parameters were drawn from random variables [32–36]. To consider the possibility of sampling bias from all possible samples, we assessed the studies for publication bias. First, we visually inspected the studies for symmetry of the funnel plot (supplemental figure A). Second, we ran the Tweedie’s Trim and Fill test to ensure that the publication bias could not reverse our estimate of the effect sizes. [31]

## Results

Thirteen articles were included in the qualitative synthesis portion of this review and six studies were included for the quantitative synthesis. Table 1 summarizes study information on study sample, design, and assessments. Table 2 summarizes study results and relevant information.

## Qualitative Synthesis

### Study Characteristics

The total number of participants of the 14 studies included in the qualitative synthesis was 11,295 individuals ranging from 13 to 4,712. The overall demographics of this review are presented in Table 1. The countries included the United States (n = 5) [37–41], Australia [42], France [43], Italy [44], Netherlands [45], South Korea [46], and China [47–49]. A study done by Winer and colleagues collected data from participants in multiple countries, including the United States, Canada, Australia, and Japan [50]. All of the studies used a cross-sectional study design [37–50].

The mean age of the samples ranged from 61.6 to 75.7. Data from the studies included study subjects, and most studies had specific inclusion criteria for the mean age and cognitive status [37–50]. All studies only included individuals who were cognitively healthy without any neurological or untreated psychological conditions or certain health conditions that may affect sleep and Aβ. Exclusion criteria for all studies in our analysis were low cognition or markers associated with cognitive impairment such as lesions, stroke, or neurological disorders [37–44, 46–50], other major illnesses [38, 40, 44, 47, 49], and drugs that are active in the CNS [38].

### Quality Assessment

**Figure 2:**
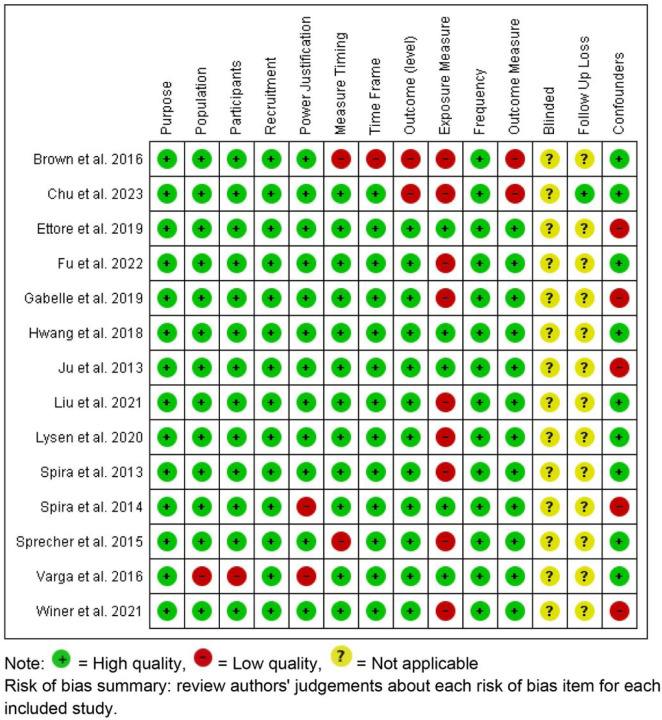
Risk of bias summary

Figure 2 illustrates the assessment of the risk of bias categories. Among the 13 observational studies, three had a moderate to high risk of bias due to measurement timing, the outcome measure, exposure measure, or the population and participants. Two of the articles had a risk of bias related to a small sample size and population without any power justification. Two of the studies reported a high risk of bias related to the measurement timing, timeframe, and outcome and exposure variable. Eight of the articles had a risk of bias related to the exposure measure using a self-report sleep question or questionnaire. Five articles had a risk of bias due to the limited number of confounding variables.

### Sleep Measures

Both subjective and objective sleep measures were used in the reviewed studies (Table 1). Five studies used objective measurements, including polysomnography (PSG) [38, 40] and actigraphy [37, 44, 46]. Overnight data were collected in studies with PSG. Of the three articles assessing sleep duration and sleep efficiency with actigraphy, two studies used Actiwatch 2 (Phillips Respironics) [37, 46]. Ettore et al. (2019) used a three-axis accelerometer (GT3X+, Actigraph Corp, Pensacola, FL). All actigraphy data were collected in 60-second epochs, and the duration of the actigraphy recording ranged from 6 to 14 days.

Eight of the reviewed studies used subjective measurements including the Pittsburgh Sleep Quality Index (n = 4) [42, 45, 47–49], a standardized interview to assess sleep duration (n = 3) [39, 43, 50], and the sleep scale in the Medical Outcomes Study (n = 1) [41]. The interviews included questions related to the duration of nighttime sleep, daytime sleep, total sleep time (daytime and nighttime sleep), and sleep efficiency [43]. Four articles used sleep duration as a categorical variable. However, Spira et al. (2013) coded the categorized sleep variable in a continuous manner by coding 0 for sleep duration longer than 7 hours a night, and 3 for sleep duration of 5 hours or less. The other two papers by Gabelle et al. (2019) and Winer et al. (2021) categorized sleep duration as shorter (6 hours or less), normal (6–7h and 6–8h), and longer (≥ 7 h or ≥ 9 h) sleep duration. Chu et al. (2023) categorized the sleep duration by interquartile range (< 5 h, ≥ 5 h to < 6 h, ≥ 6 h to < 7 h, ≥ 7 h to < 8 h, > 8 h), then used dichotomous variable of sleep duration less than or equal to 8 and greater than 8 hours. The time window for sleep duration assessment varied from 1 day to four weeks. Ten articles treated the sleep duration variable as a continuous variable.

### Amyloid Measures

The reviewed studies used a variety of Aβ measures (Table 1). The 13 studies measured Aβ concentration from bodily fluids including CSF (n = 3) [37, 40, 48] and peripheral blood (n = 2) [45, 47]. CSF was obtained by lumbar puncture the morning after overnight fasting (from 8am to 10am) [37] or CSF samples collected between 11:00–13:00 [40]. In three studies that tested peripheral blood, samples were collected in the morning after overnight fasting [45, 47], and were processed using plasma for further analysis. These studies measured Aβ_40_ and Aβ_42_ and assessed different combined ratios (e.g., Aβ_42,_/Aβ_40_, P-tau/Aβ_42_, T-tau/Aβ_42,_ NFL/Aβ_42_)[37, 40, 45, 47, 48] by an enzyme-linked immunosorbent assay (ELISA) [37, 40, 47–49] or by Simoa [45].

Nine studies measured Aβ using a PET scan [38, 39, 41–44, 46, 49, 50]. These studies obtained the amyloid deposit using two tracers: five studies used Carbone11 labeled Pittsburgh compound B (11C-PiB) [38, 41, 42, 46], six studies used fluorine 18 (18F) labeled tracers including 18F-florbetapir [39, 42–44, 49, 50], and one study used 18F-flutemetamol [42]. Brown et al. (2016) utilized data using three tracers: [C-11] PiB, and 18F-florbetapir, and 18F-flutemetamol [42].

Quantitative assessment was done using standardized uptake value ratios (SUVr) in four studies [42–44, 46], and the distribution volume ratio (DVR) in three studies [38, 41, 50]. Three studies with PET brain imaging used a cutoff to determine amyloid positivity. Ettore et al. (2019) used SUVr of 0.7918, Gabelle et al. (2019) used SUVr of 1.17, and Hwang et al. (2018) used SUVr of 1.21. Ju et al. (2013) used CSF Aβ_42_ of 500pg/ml for the cut off.

Table 2 presents the covariate adjustments used in the statistical analyses of the studies. In general, the studies accounted for age and sex, except for Gabelle et al. (2019). Race and ethnicity were accounted for in two studies [39, 50]. Nine studies controlled for APOEε4 allele [37, 39–41, 43, 46–49]. Several studies also adjusted for education [40, 42, 45, 47, 48, 50]. Clinical factors that were accounted for in the studies included depression [39, 42–44, 46, 47, 49], and cognition status measured by the mini-mental state examination (MMSE) [38, 41, 42, 45, 47] or Montreal Cognitive Assessment (MOCA)[49]. Body mass index was a common lifestyle covariate considered in several studies [38, 41, 42, 45, 47, 49]. Other lifestyle covariates included hypertension [45, 47–49] and diabetes [45, 47–49]. Sleep and circadian rhythm variables were also included such as slow wave sleep duration [40], sleep disturbance[49], sleep apnea [45], or sleep medication [39]. Other factors included family history of AD, alcohol and caffeine consumption [45, 48, 49], cholesterol levels [45, 47], and exercise [47].

### Association Between Exposure And Outcomes

Table 2 describes the findings of the reviewed studies for the qualitative synthesis. Five of the 14 articles [39, 47–50] found that shorter sleep duration was associated with higher Aβ. However, three of the studies reported the reverse association between sleep duration and PET-measured global and regional Aβ burden. Winer et al., (2021) found that self-reported shorter sleep duration was associated with greater 18 F-florbetapir-PET brain imaging derived DVR Aβ burden (β = − 0.01; p = .005). Spira et al. (2014) also reported that shorter sleep duration was associated with greater Aβ burden, measured by mean cortical [C-11] PiB PET derived DVR (cDVR; B = 0.08, p = 0.005) and precuneus DVR (β = 0.11, p = 0.007). Longer total sleep time was associated with reduced 18 F-florbetapir-PET brain imaging derived SUVr global Aβ (β =−0.005; p = .03), reduced medial orbitofrontal Aβ (β =−0.009; p < .001), and reduced anterior cingulate Aβ (β = −0.011; p < .001). Sleep duration longer than 8 hours is associated with having higher amyloid burden compared to sleep duration shorter or equal to 8 hours (Odds Ratio = 4.167; p = 0.020)[49]. In addition to studies using PET, findings from a Chinese sample by Liu et al. (2021) also found that shorter sleep duration was associated with higher plasma Aβ_42_ (β = 0.495, p = 0.021) and Aβ_42_/Aβ_40_ ratio (β = 0.101, p < 0.001)[47]. Fu et al. (2021) also found a non-linear relationship indicating a decrease in CSF Aβ42 with shorter or longer sleep duration, with the extreme point being 6.23 hours of sleep [48].

### Quantitative synthesis

**Figure 3:**
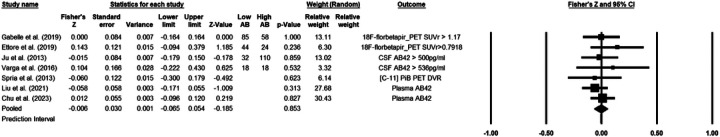
Forest plot of overall Fisher’s Z score for association between sleep duration with Amyloids beta.

Six eligible articles were used for the quantitative synthesis because the remaining studies were not included due to a lack of reporting Pearson’s correlation or not having a mean sleep duration for the amyloid beta categories. Figure 3 and Table 3 are summaries of the quantitative synthesis of the articles. The findings demonstrated that the average association between sleep duration and Aβ was not statistically significant (Fisher’s Z = −0.006, 95% CI= −0.065 ~ 0.054). The Z-value is −0.185 with p = 0.853. Using a criterion alpha of 0.050, we cannot reject the null hypothesis. As in Table 3, for heterogeneity, our results indicate a Q-value of 3.101 with 6 degrees of freedom, indicating that the amount of between-study variance in the observed effect is less than we expected based on sampling error alone. The I^2^ statistic is 0%, indicating that 0% of the variance in observed effects reflect the variance in true effects rather than sampling error. Tau reflects the standard deviation of true effect size, which is 0.000 in Fisher’s Z units.

As shown in the standard error funnel plot by Fisher’s Z (supplemental Figure A), the plot is slightly asymmetric, indicating that there could be minor publication bias from the included studies. This might be due to either our inability to identify studies with non-significant finings or failing to report non-significant findings [31]. We ran the Tweedie’s Trim and Fill method, which demonstrated that even if we remove one study, the effect size remains statistically insignificant (Fisher’s Z= −0.0056, 95% CI= −0.0649 ~ 0.0537). This finding may imply that some of the articles might not have presented the findings due to non-significant results.

## Discussion

Our review synthesized fourteen studies for the qualitative synthesis and seven studies for the quantitative synthesis focusing on sleep duration and Aβ. Using seven eligible articles, our quantitative synthesis demonstrated that the average association between sleep duration and Aβ was not statistically significant (Fisher’s Z = −0.013, 95% CI= −0.084 ~ 0.058). This review suggests that caution should be taken when considering sleep duration as the primary factor for Aβ levels. The studies used subjective questionnaires or questions, PSG, or actigraphy to measure sleep duration. Aβ was measured using PET CT, CSF, or serum. There are quality concerns about a few studies due to the small sample size, timing of the measures, limited use of comprehensive measurements, and confounding variables.

Although not all 14 qualitative studies we reviewed demonstrated statistically significant associations between sleep duration and Aβ, five studies showed that shorter sleep duration was associated with greater Aβ, but one study reported sleep great than 8 hours is associated with Aβ burden. The quantitative synthesis revealed an effect size of −0.006, but it was not statistically significant, indicating that sleep duration may not be a primary factor in Aβ accumulation. Alternatively, these results may be due to moderators (i.e., APOE4, sex, age, family history, or unmeasured moderators), heterogenous outcome types of Aβ that may not provide consistent ideas, or publication bias due to insignificant results not being favorable for publication. Prior research has demonstrated that chronic sleep restriction or deprivation of slow wave sleep can alter the diurnal fluctuation of CSF Aβ levels [24, 25, 51, 52]. Sleep deprivation may also impair human memory consolidation, in part by reducing the synthesis of proteins needed to support synaptic plasticity [14, 53–55]. In a meta-analysis by Wu and colleagues (2018), the authors suggest that there is a U-shaped relationship between sleep duration and cognitive disorders. Compared to the reference group (7–8 hours per day), individuals with a short or long duration had a higher risk of developing cognitive disorders, such as Alzheimer’s disease or dementia [56]. Both a shorter sleep duration (< 7 hours / night) and poor subjective sleep quality are important for cognitive function [57] or brain structures and functions [58, 59]. However, more studies in this field need using larger sample sizes, a prospective design, and publication of magnitude of correlations of null results can shed light on the true relationship between sleep duration and Aβ.

There was considerable heterogeneity in the methods used in the reviewed studies to conclude the relationship between sleep duration and Aβ burden. Although self-report sleep duration may not provide an accurate estimate of sleep duration, eight of thirteen reviewed studies used self-report measures, such as Pittsburgh sleep quality index (n = 6), medical outcomes study sleep scale (n = 1), or single question (n = 3) to assess sleep duration. The interview question in most studies directly asked participants about the average total number of hours of sleep, but these studies did not ask about the time window of habitual sleep or other dimensions. The time window of sleep duration assessments varied from 1 night with polysomnography to 4 weeks using Pittsburgh sleep quality index. Future studies are needed to capture habitual sleep duration using an actigraphy with a verification with sleep diary for 7 to 14 days [60]. The use of a polysomnography would also provide insights on the structure of sleep and sleep disorders [61–63].

Both the measures and the types of data were different in the studies. The majority of the studies used sleep duration as a continuous format using multivariate linear regression models. Winer et al. (2021) and Liu et al. (2021) used a categorial variable in the model and provided insights on the dose-dependent relationship between sleep duration and Aβ[47, 50]. Both studies indicated that a shorter sleep duration compared to the standard 7–8 hours a night or 7 hours or more sleep is associated with greater Aβ. Among the reviewed studies, Fu and colleagues reported a non-linear relationship between sleep duration and CSF measured Aβ_42_ demonstrating lower Aβ_42_ values for shorter or longer sleep and the highest for 6.23 hours [48]. Future studies using comprehensive and accurate assessment of sleep as well as a non-linear model would provide deeper insights on recommendations for sleep duration.

In addition to the considerable variability in sleep measurements, Aβ was also measured in different ways: PET to quantify the Aβ burden, CSF, and serum sample. Studies using PET used different tracers (e.g., 11C-PiB, 18F-florbetapir, 18F-flutemetamol) as well as different quantification methods. Most studies focused on global Aβ burden in brain, but assessing both the overall levels of Aβ in PET as well as specific regional deposition could help us understand areas of the brain that may be affected more than other areas. This variability across the measurements prevented us from conducting a meta-analysis and drawing strong conclusions. However, it is promising in the current field of science to review data across different measures of Aβ accumulation. Although AD can be diagnosed at an autopsy [64], the US National Institute on Aging and Alzheimer’s Association have suggested using Aβ as well as tau and neurodegeneration to de ne and diagnose AD in both symptomatic and a symptomatic stages [65]. Increased accessibility to biomarkers and the potential for blood biomarkers or additional biomarkers in addition to Aβ would provide further information about the underlying disease processes in the future.

In addition to sleep duration, other sleep dimensions could be important factors for Aβ accumulation. First, reduced sleep efficiency could be a critical factor associated with Aβ accumulation. Of the reviewed studies, two reported that lower sleep efficiency was associated with Aβ burden. Ettore et al. (2019) found that an increase in sleep efficiency was associated with a 41% reduction of Aβ positivity (Odds Ratio = 0.59, < 0.001). Ju et al. (2013) reported that individuals with a low CSF Aβ_42_ level (≤ 500 pg/mL), which is indicative of amyloid deposition in the brain, had worse sleep efficiency than those with a normal CSF Aβ_42_ level (80.4% vs. 83.7%, p = 0.08), although sleep duration was not statistically significant. The reviewed studies also identified a positive link between Aβ and different sleep characteristics including less adequate sleep, more sleep problems, and greater somnolence based on participants’ self-reported perceptions [41], sleep quality [39], frequent napping [37], longer sleep latency [42, 44], greater sleep fragmentation [44], a higher apnea hypopnea index, and slow wave sleep time [38, 40]. These results may indicate that different dimensions of sleep could contribute more to Aβ burden than quantity of sleep.

The reviewed studies accounted for various demographic and clinical confounders in the multivariate models. Most of the reviewed studies accounted for age and sex, which are well-known confounders [10, 66–68]. Individuals with sleep disorders, underlying health or psychological conditions, medications, genetic factors, social determinants, high fat diets, and physical activity have different sleep quantity and quality [69–71]. These factors may also increase the amount of Aβ accumulation [72–79]. These confounding factors may play critical roles in determining the association between sleep duration and Aβ burden. However, the studies used a cross-sectional design, which prevents us from drawing causal relationships. Specifically, some of the studies did not measure sleep and Aβ burden in a similar time period, and thus the results may not reflect a direct link between the two factors. Although researchers have speculated that the relationship between sleep and AD pathology could be bidirectional, there is limited evidence to support the longitudinal relationships [21, 80].

The strength of this review is that we examined current evidence related to sleep duration and amyloid burden. However, there are a few limitations. First, the study did not test for moderating effects of age, sex, APOE4 status, or sleep efficiency. Second, the current study only included publications written in English even though some important findings may have been published in different languages. Third, this review focused only on sleep duration even though other specific sleep characteristics could have more influence on Aβ pathology.

## Conclusions

The results of this systematic review suggest that previous studies that have demonstrated an association between sleep duration and Aβ accumulation should be understood cautiously. Researchers would greatly benefit from more studies using a longitudinal design, comprehensive sleep measure, broad range of biomarkers, and larger sample sizes to advance our scholarly understanding of the relationship between sleep and AD.

## Figures and Tables

**Table 1:** Study characteristics

First author (year)	Study Design	Sample name, country	Sample size	Mean age± SD (years)	Exposure Assessment	Time window of Sleep duration	Outcome Assessment	Covariates
Ju (2013) [37]	Cross-sectional	Washington University Knight Alzheimer’s Disease Research Center & Adult Children Study, USA	142	65.6 ±8.2	Actigraphy (Actiwatch 2, Phillips Respironics)	2 weeks	CSF measured Aβ42	Age, sex, APOEε4 allele
Spira (2013) [39]	Cross-sectional	From other studies or the community in Baltimore, MD, USA	13	Normal = 69.4 ±5.6 ;MCI = 75.2 ±11.3	PSG	2 nights (The first night was for adaptation. only second night data were used.)	18 F-florbetapir-PET Brain Imaging derived DVR	Age, sex, BMI
Spira (2014) [38]	Cross-sectional	Baltimore Longitudinal Study of Aging, USA	70	78.2 ±7.9 when they completed PiB PET, and 76.4 ± 8.0 (range 53–91) when they completed sleep measures	Standardized interview of mean number of hours of sleep obtained each night during the prior month using the following response options: “more than 7”; “more than 6, up to 7”; “more than S, up to 6”; or “5 or fewer.”	4 weeks	[C-11] PiB PET derived DVR	Age, sex, race, APOE ε 4, depressive symptoms, BMI, cardiovascular or pulmonary disease, and use of sleep medication (any vs. none)
Sprecher (2015) [41]	Cross-sectional	Wisconsin Registry for AD (WRAP), USA	98	Age at PiB PET scan = 62.4 ±5.7; Age at sleep assessment 63.0 ±5.6	Self -report Medical Outcomes Study Sleep Scale	4 weeks	[C-11] PiB PET derived DVR	Age, sex, APOE ε4, family history of Alzheimer’s Disease, BMI
Brown (2016) [42]	Cross-sectional	Australian Imaging, Biomarkers and Life- style (AIBL) study of aging, Australia	184	75.5 ±6.1	PSQI	4 weeks	[C-11] PiB PET derived SUV, 18F-flutemetamol (FLUTE) derived SUVr, and 18F-florbetapir (FBP) derived SUVr.	Age at PET scan, sex, years of education, depressive symptoms, time between sleep assessment and PET scan, Aβ burden, MMSE, BMI
Varga (2016) [40]	Cross-sectional	Community dwelling older adults from New York City Area, USA	36	66.8 ±8.2	PSG	1 night	CSF measured Aβ40 and Aβ42	Age, sex, APOE ε4, education, SWS duration, %TST in SWS, mean SWS bout length, total SWA, SWA in NREM cycles 1–4, cerebrospinal fluid biomarkers, medial prefrontal cortex volume
Hwang (2018) [46]	Cross-sectional	Brain Aging Study, Korea	133	68.05 ± 7.68	Actigraphy (Actiwatch 2, Phillips Respironics, Murrysville, PA)	8 days	[C-11] PiB PET derived SUVr	Age, sex, depression symptoms, APOE ε4, selected actigraphic sleep and circadian variables
Gabelle (2019) [43]	Cross-sectional	MAPT-AAV45 sleep ancillary study, France	143	median: 73 [70–85]	Standardized interview	4 weeks	18 F-florbetapir-PET Brain Imaging derived SUVr	APOE ε4, depression
Ettore (2019) [44]	Cross-sectional	INveStIGation of Alzheimer’s Predictors in Subjective Memory Complainers (INSIGHT)- preAD Study, Italy	68	76.67 ± 3.52	Actigraphy (GT3X)	7 days	18 F-florbetapir-PET Brain Imaging derived SUVr	Age, sex, depression, MMSE
Lysen [(4250]20)	Cross-sectional	prospective Rotterdam Study cohort, Netherlands	4712	72 ±8	PSQI	8 days	Plasma measured Aβ40 and Aβ42	Age, sex, education, presence of selfreported paid employment, time interval between measurements of sleep and biomarker, possible sleep apnea, batch number of biomarker analysis, habitual alcohol consumption, smoking status, BMI, hypertension, diabetes, T-cholesterol previous history of heart disease
Winer Í2021)	Cross-sectional	Anti-Amyloid Treatment in Asymptomatic Alzheimer Disease (A4) study, US, Canada, Australia, and Japan.	4417	71.3 ±4.8	Standardized interview question of “average total number of hours slept at night”	N/A	18 F-florbetapir-PET Brain Imaging derived dVr	Age, sex, years of education, self-identified race/ethnicity, number of APOE ε2 alleles, and number of APOE ε4 alleles
Liu (4021)	Cross-sectional	Cognitive Disorders Clinics in the First People’s Hospital of Foshan and communities, China	305	69.07 ± 6.37	PSQI	4 weeks	Plasma measured Aβ40 and Aβ42	Model 1: Age, sex, education Model 2: APOE ε4, depressive symptoms, MMSE, BMI, exercise frequency, diabetes, hypertension, triglyceride, fasting blood glucose
Fu (2021) [48]	Cross-sectional	Chinese Alzheimer’s Biomarker and Lifestyle study, China	974	61.6 ± 10.3	PSQI	4 weeks	CSF measured Aβ49 and Aβ42, phosphorylated tau (P-tau)	Age, sex, education, ApOE ε4 status, hypertension, diabetes, coronary heart disease, stroke, smoking and drinking
Chu (2023) [49]	Cross-sectional	Community dwelling older adults from Shanghai Sixth People’s Hospital Affiliated to Shanghai Jiao Tong University School of Medicine	335	64.4 ±7.8	PSQI	4 weeks	18 F-florbetapir-PET Brain Imaging derived dVr and plasma measured Aβ49 and Aβ42	Age, sex, education, bMi, smoking, alcohol consumption, APOE ε4 status, Chinese version of Montreal Cognitive Assessment-Basic, hypertension, diabetes, hyperlipidemia, coronary artery disease, Aβ42/40, neurofilament light chain, sleep duration >8 hours, sleep disturbance

Abbreviations: PiB = Pittsburgh Compound B; PET = positron emission tomography; DVR = distribution volume ratio; BMI = body mass index; CSF = cerebrospinal fluid; PSG = Polysomnography; PSQI = Pittsburgh Sleep Quality Index; SUVr = standardized uptake value ratio; MMSE = Mini Mental State Examination; TST = total sleep time; SWS = slow wave sleep; SWA = slow wave activity; NREM = non-rapid eye movement.

**Table 2:** Study results

Sleep Duration and Amyloid				
First author(year)	Exposure categories	Amyloid measure type (serum, CSF, PET)	Outcome Definition	Results
Ju (2013) [37]	Continuous	CSF measured Aβ42	Aβ 42>500pg/ml	No significant association between actigraphy measured sleep duration and CSF Aβ42 levels.
Spira (2013) [39]	Continuous	18 F-florbetapir-PET Brain Imaging derived DVR	Continuous	No significant association between sleep duration and Aβ deposition.
Spira (2014) [38]	Continuous (“more than 7”; “more than 6, up to 7”; “more than 5, up to 6”; or “5 or fewer” were coded in 0 to 5)	[C-11] PiB PET derived DVR	Continuous	Shorter sleep duration was associated with greater β-amyloid burden, measured by mean cortical DVR (cDVR; B = 0.08, 95% confidence interval (CI) 0.03, 0.14, p = 0.005) and precuneus DVR (B = 0.11, 95% CI 0.03, 0.18, p = 0.007)
Sprecher (2015) [41]	Continuous	[C-11] PiB PET derived DVR	Continuous	No significant association between sleep duration and amyloid burden.
Brown (2016) [42]	Continuous	[C-11] PiB PET derived SUV 18F-flutemetamol (FLUTE) derived SUVr, and 18F-florbetapir derived SUVr.	Continuous	No significant association between sleep duration and brain Aβ burden.
Varga (2016)[40]	Continuous	CSF measured Aβ40and Aβ42	Continuous	No significant association between total sleep time and CSF Aβ42.
Hwang (2018) [46]	Continuous	[C-11] PiB PET derived SUVr	Amyloid positive: SUVr> 1.21	No significant association between sleep duration and Aβ positivity.
Gabelle (2019) [43]	Sleep duration (as a continuous variable, and categorized into < 6; 6–7; ≥7 h per night); sleep efficiency (less than 82.35; 82.35–93.75; more than 93.75)	18 F- florbetapir -PET Brain Imaging derived SUVr	Amyloid positive: SUVr> 1.17	No significant association between nighttime sleep duration (as a continuous variable or categorized into < 6; 6–7; ≥7 h per night) and Aβ positivity.
Ettore (2019) [44]	Continuous	18 F-florbetapir-PET Brain Imaging derived SUVr	Amyloid positive: SUVr>0.7918	No significant association between positive Aβ status and total sleep time.
Lysen (2020) [45]	Continuous	Plasma measured Aβ40 and Aβ42	Continuous	No significant association between self-reported sleep duration and plasma β-amyloid 40 and β-amyloid 42.
Winer (2021)[50]	Grouped by short sleep duration: less than or equal to 6 hours, normal sleep duration: 7–8 hours, and long sleep duration: more than or equal to 9 hours	18 F-florbetapir-PET Brain Imaging derived DVR	Continuous	Self-reported shorter sleep duration was linearly associated with higher Aβ burden (β [SE] = − 0.01 [0.00]; P = .005). No difference in Aβ was found between long and normal sleep duration groups (β [SE] = 0.00 [0.01]; P = .99).
Liu (2021) [47]	Sleep duration (more than 7; 6–7; less than 6); Sleep efficiency(more than 85%; 75–84%; 65–74%; less than 65%)	Plasma measured Aβ40 and Aβ42	Continuous	Sleep duration was negatively associated with plasma Aβ42 level (β = −0.267, 95% CI - 0.450 ~ - 0.084, p = 0.005) and Aβ42/Aβ40 ratio (β = −0.058, 95% CI − 0.077 ~− 0.039, p< 0.001).
Fu (2021) [48]	Continuous	CSF measured Aβ40 and Aβ42	Continuous	Sleep duration was significantly associated with plasma Aβ42 level (β = 2.71 E-03; P = p< 0.01). *Nonlinear relationships
Chu (2023) [49]	Sleep duration greater than 8 hours (yes/no) for multivariate analysis and continuous for correlational analysis.	18 F-florbetapir-PET Brain Imaging derived DVR and plasma measured Aβ40 and Aβ42	Amyloid positive based on visual rating and continuous for correlational analysis	Sleeping more than 8 hours were associated with developing Amyloid positive (OR = 1.992, p = 0.033). Sleep duration was not associated with Aβ42, Aβ40, or Aβ40/42.

Abbreviations: PiB = Pittsburgh Compound B; PET = positron emission tomography; DVR = distribution volume ratio; CSF = cerebrospinal fluid; SUVr = standardized uptake value ratio.

**Table 3:** Overall Fisher’s Z score for association between sleep duration with Amyloids beta.

Model		Effect size and 95% confidence interval					Test of null (2-Tail)		Prediction Interval		Between-study		Other heterogeneity statistics			
Model	Number Studies	Point estimate	Standard error	Variance	Lower limit	Upper limit	Z-value	P-value	Lower limit	Upper limit	Tau	TauSq	Q-value	df (Q)	P-value	I-squared
Random effects	7	−0.006	0.030	0.001	−0.065	0.054	−0.185	0.853			0	0	3.101	6	0.796	0.000

## Data Availability

Data will be available upon request to the corresponding author.

## Abbreviations

Aβ: Amyloid β
AD: Alzheimer’s disease
CSF: cerebrospinal fluid
CMA: Comprehensive Meta-Analysis
DVR: distribution volume ratio
MMSE: the mini-mental state examination
MOCA: Montreal Cognitive Assessment
PET: positron emission tomography
PRISMA: Preferred Reporting Items for Systematic Reviews and Meta-Analyses
PSG: polysomnography
SUVr: standardized uptake value ratios
11C-PiB: Carbone11 labeled Pittsburgh compound B
18F: fluorine 18
